# P-840. Optimizing Diagnostic Stewardship: Reducing Routine AFB and Fungal Cultures in Orthopedic Surgical Specimens

**DOI:** 10.1093/ofid/ofaf695.1048

**Published:** 2026-01-11

**Authors:** Whitney Miller, Sean Higinbotham, Jessica Churchill, Kristine Hoffman, Margaret M Cooper, Michael L Wilson, Timothy C Jenkins

**Affiliations:** Denver Health, Denver, CO; Denver Health, Denver, CO; Denver Health, Denver, CO; Denver Health, Denver, CO; Denver Health, Denver, CO; Denver Health and Hospital Authority, Denver, Colorado; Denver Health, Denver, CO

## Abstract

**Background:**

Fungal and mycobacterial cultures are frequently ordered on orthopedic operative specimens but are low yield and rarely impact patient management. The objective of this study was to evaluate the effects of an intervention to reduce unnecessary fungal and mycobacterial cultures during orthopedic surgeries.

**Figure:**
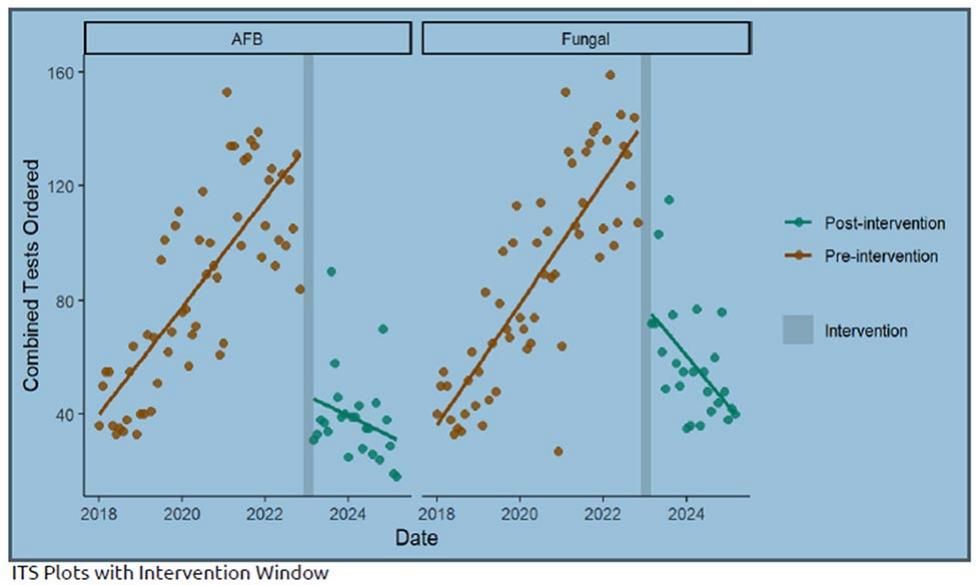
Combined AFB/Fungal Cx Pre and Post Intervention

**Figure:**
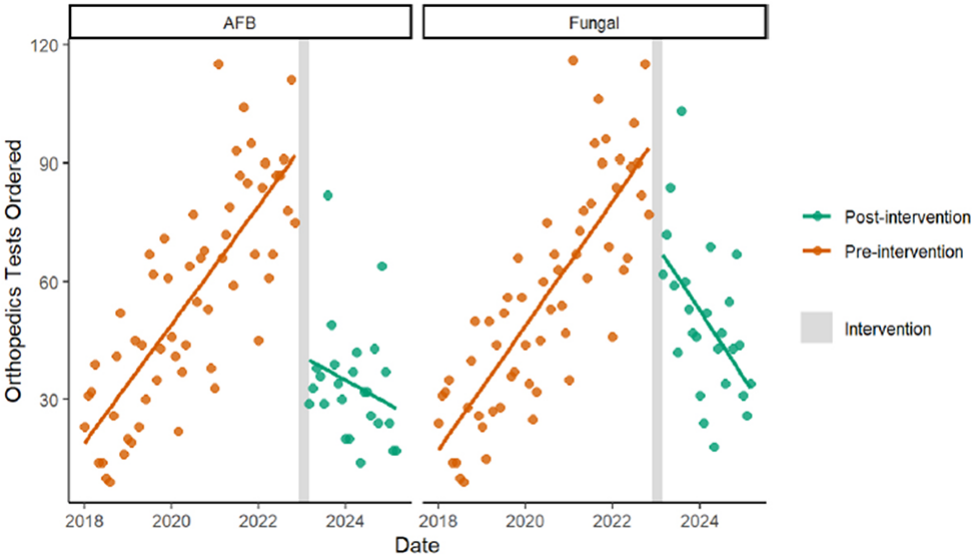
Orthopedics AFB/Fungal Cx Pre and Post Intervention

**Methods:**

This was a quasi-experimental study of patients undergoing an orthopedic or podiatric surgical procedures at a 550-bed academic, safety-net hospital. The pre-intervention period was 1/2018 to 11/2022; the post-intervention period was 3/2023 to 3/2025. The intervention (12/2022 – 2/2023) included education to Orthopedic Surgeons, Podiatrists, and operating room nursing staff to discontinue routine use of fungal and mycobacterial cultures in favor of targeted use when clinical suspicion for fungal or mycobacterial infection was higher. Interrupted time series analysis was used to evaluate changes in fungal and mycobacterial cultures performed before and after the intervention. Secondary outcomes included the annual number of tests averted, cost savings, and change in laboratory personnel time.

**Figure:**
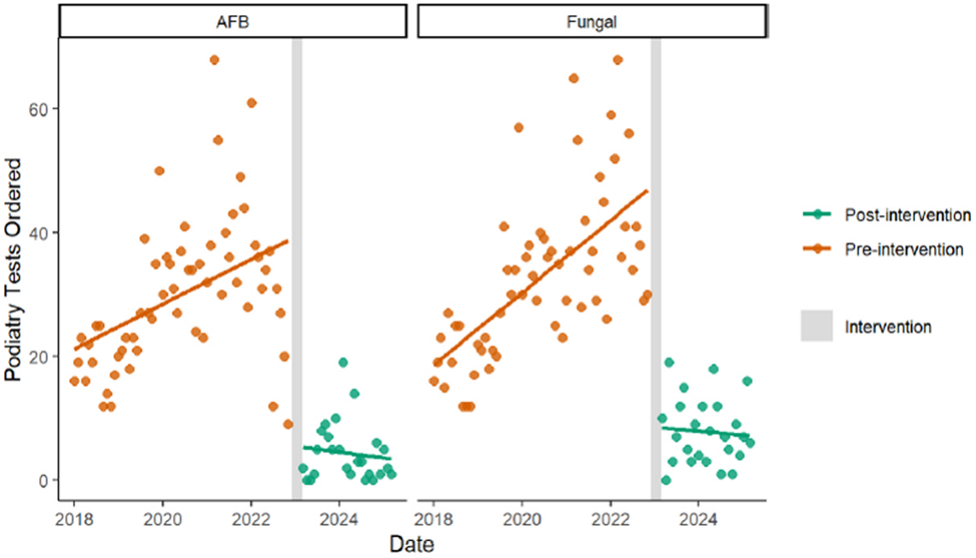
Podiatry AFB/Fungal Cx Pre and Post Intervention

**Results:**

During the pre-intervention period, there were significant increasing trends in the number of fungal cultures (1.3 tests/month, p< .001) and mycobacterial cultures (1.0 tests/month, p< .001) performed (Figure 1). Immediately after the intervention, there were significant reductions (intercept changes) in fungal cultures (-62.5 tests, p< .001) and mycobacterial cultures (-84.9 tests, p< .001). During the post-intervention period, there were significant declining trends in fungal cultures (-5.4 tests/month, p< .001) and mycobacterial cultures (-5.1 tests/month, p< .001). The results were similar when stratified by orthopedic (Figure 2) versus podiatric surgeries (Figure 3). The intervention averted 976 fungal cultures and 975 mycobacterial cultures per year, decreased laboratory costs by $53,362 per year, and decreased laboratory personnel time by 894 hours per year.

**Conclusion:**

An intervention to promote targeted rather than routine use of fungal and mycobacterial cultures in orthopedic surgeries significantly reduced the number of cultures performed resulting in substantial cost savings and improved efficiency.

**Disclosures:**

All Authors: No reported disclosures

